# Antimicrobial use and *Escherichia coli* resistance patterns in Hungarian pig farms: a data-driven farm-level analysis

**DOI:** 10.1038/s41598-026-43008-7

**Published:** 2026-03-03

**Authors:** Krisztián Vribék, Máté Farkas, Szilveszter Csorba, Miklós Süth, László Gombos, Ádám Kerek, Zoltán Somogyi, Ákos Jerzsele, Zsuzsa Farkas

**Affiliations:** 1 Department of Digital Food Science, Institute of Food Chain Science, University of Veterinary Medicine, Budapest, H-1078 Hungary; 2 National Laboratory of Infectious Animal Diseases, Antimicrobial Resistance, Veterinary Public Health and Food Chain Safety, University of Veterinary Medicine Budapest, Budapest, H-1078 Hungary; 3 Institute of Food Chain Science, University of Veterinary Medicine, Budapest, H-1078 Hungary; 4 Wittmann Antal Multidisciplinary Doctoral School of Plant, Animal, and Food Sciences, Széchenyi István University, 2 Vár Square, Mosonmagyaróvár, H-9200 Hungary; 5 Department of Pharmacology and Toxicology, University of Veterinary Medicine, Budapest, H-1078 Hungary

**Keywords:** Antimicrobial use, AMR, Escherichia coli, MIC, Real-world data, Swine production, Diseases, Microbiology

## Abstract

Antimicrobial resistance (AMR) poses a critical challenge to both human and veterinary medicine, with pig production recognized as one of the major contributor due to intensive antimicrobial usage (AMU). This study aimed to explore the relationship between AMU and AMR patterns of *Escherichia coli* isolated from commercial pig farms, using data-driven analytical methods. Farm-level records were harmonized with microbiological data from 203 isolates collected in December 2023 across four Hungarian farms. AMU was summarized over 3-, 6-, 9-, and 12-month retrospective windows and expressed in modified population-corrected units, while AMR was quantified as mean minimum inhibitory concentration (MIC) and AMR rate under epidemiological and clinical breakpoints. The results revealed substantial variation in AMU among farms, with amoxicillin predominating across timeframes. Farm-specific comparisons indicated that higher AMU may not always coincide with elevated resistance levels, and data analysis did not consistently identify a direct association between use and resistance at the individual farm level, which warrants further investigation in larger datasets. Correlation analyses identified strong intra-class relationships among β-lactams and fluoroquinolones, as well as a cross-class linking, suggesting concurrent selection pressures. Overall, the integration of AMU and AMR data demonstrated the feasibility of farm-level surveillance for AMR modelling and provides a foundation for future predictive systems to support antimicrobial stewardship in livestock production.

## Introduction

Antimicrobial resistance (AMR) is one of the most pressing global health threats of the 21 st century, jeopardizing the effectiveness of antimicrobials in treating infectious diseases in both human and veterinary medicine. The World Health Organization has recognized AMR as a top-ten global public health threat due to its contribution to prolonged illnesses, increased mortality, and escalating healthcare costs^1,2^. AMR develops primarily through genetic mutations and the horizontal transfer of resistance genes among bacteria, allowing them to survive even greater dosage of antimicrobial exposure^3^. While AMR is a natural evolutionary process^4^, it has been significantly accelerated by the misuse and overuse of antimicrobials across multiple sectors. In human medicine, antimicrobials are often prescribed inappropriately, for example to treat viral infections or without confirming bacterial etiology. These irresponsible practices among others foster the selection of resistant strains^5^. In veterinary settings, especially in intensive livestock production systems, antimicrobials have historically been used not only for therapeutic purposes but also prophylactically and for growth promotion^6^. Such practices have been particularly widespread in pig farming, contributing to the emergence and dissemination of resistance genes in both animal and environmental reservoirs^7^. Therefore, AMR in swine production is a growing concern as well, especially regarding pathogen such as *Escherichia coli*. This bacterium is a primary cause of post-weaning diarrhea and oedema disease in piglets, leading to significant economic losses^8^. Studies have shown that *E. coli* isolates from pigs often exhibit resistance to commonly used antimicrobials, including aminoglycosides, tetracyclines, and β-lactams, primarily due to the widespread use of these antimicrobials in the industry^9^. This resistance complicates the management and intervention strategies of systemic infections in swine populations^10^.

Efforts to mitigate AMR in porcine pathogens include the development of targeted vaccines, stricter antimicrobial stewardship and biosecurity measures, and the implementation of alternative intervention methods^11^. Furthermore, pig farms also represent a critical interface where antimicrobial usage (AMU) data can be linked to the prevalence of AMR. The complexity of this relationship is influenced by various factors, including the type, frequency, and spectrum of antimicrobials used, animal health management practices, and biosecurity levels and even prudent lower level AMU can contribute to AMR^12^.

Several studies have demonstrated that the strength and shape of the AMU–AMR association strongly depend on how antimicrobial exposure is quantified^13,14^. Standardized indicators such as milligrams of active compound per population correction unit (mg/PCU), defined daily doses for animals (DDDvet), treatment incidence, and course-based metrics are commonly applied in surveillance and epidemiological analyses^13–15^. For example, analyses at herd and national levels have shown positive but often non-linear associations between AMU expressed in mg/PCU or DDDvet and resistance proportions in indicator and pathogenic bacteria, with class-specific differences and temporal lags between use and resistance emergence^13,15^. Furthermore, studies highlighted that β-lactams, tetracyclines, and fluoroquinolones frequently show the strongest usage–resistance correlations, while cross-class associations and persistence of resistance may reflect co-selection and shared mobile genetic elements rather than direct exposure alone^13,16^.

These resistant bacteria can be transmitted from animals to humans through direct contact, contaminated food products, and environmental pathways, underscoring the One Health dimension of AMR^17^. The global spread of resistant pathogens is further exacerbated by international travel and trade, highlighting the interconnectedness of human, animal, and environmental health^18^. Additionally, there is a clear need for harmonized AMU metrics and multi-scale analytical approaches when interpreting AMU–AMR relationships and comparing results across production systems and countries^13^. Despite the urgency of the problem, the development of new antimicrobial remains limited, partly due to reduced investment from pharmaceutical companies, creating a significant innovation gap in the antimicrobial pipeline^19^. To address AMR effectively, a multifaceted strategy is essential. This includes the development of novel antimicrobial agents, improved infection prevention measures, targeted vaccination programs, and the implementation of robust antimicrobial stewardship frameworks.

Importantly, real-time surveillance and predictive analytics are critical for tracking AMR patterns and guiding evidence-based interventions^20^. Interestingly, there are a few examples of data-driven approaches to visualizes trends and predicting AMR^21–24^ in pig farms^25^. Forecasting models have also been developed to simulate the impact of policy interventions, such as antimicrobial reduction programs, on future AMR trends^26^ or using anomaly detection and high-level indicators^27^. These tools not only help in anticipating AMR hotspots but also in optimizing treatment protocols and reducing unnecessary AMU^21^. These examples highlight the growing potential of data forecasting systems in addressing AMR, particularly when integrated into One Health frameworks that link human, animal, and environmental data sources^28^.

In this study, our primary objective was to explore farm-level relationships between AMU and phenotypic AMR in *E. coli* under real production conditions in Hungarian pig farms. Specifically, we aimed to (i) integrate routinely collected farm antimicrobial usage records with standardized MIC-based resistance data, (ii) characterize AMU patterns across multiple retrospective time windows, and (iii) assess the consistency and variability of AMU–AMR relationships at the individual farm level using descriptive and correlation-based analytical approaches. Rather than testing predefined hypotheses, this work was designed as an exploratory, data-driven analysis to evaluate whether routinely available farm data contain sufficient structure and signal to support future predictive modelling and surveillance-oriented applications.

## Results

### Antimicrobial usage of the farms

**Fig. 1:**
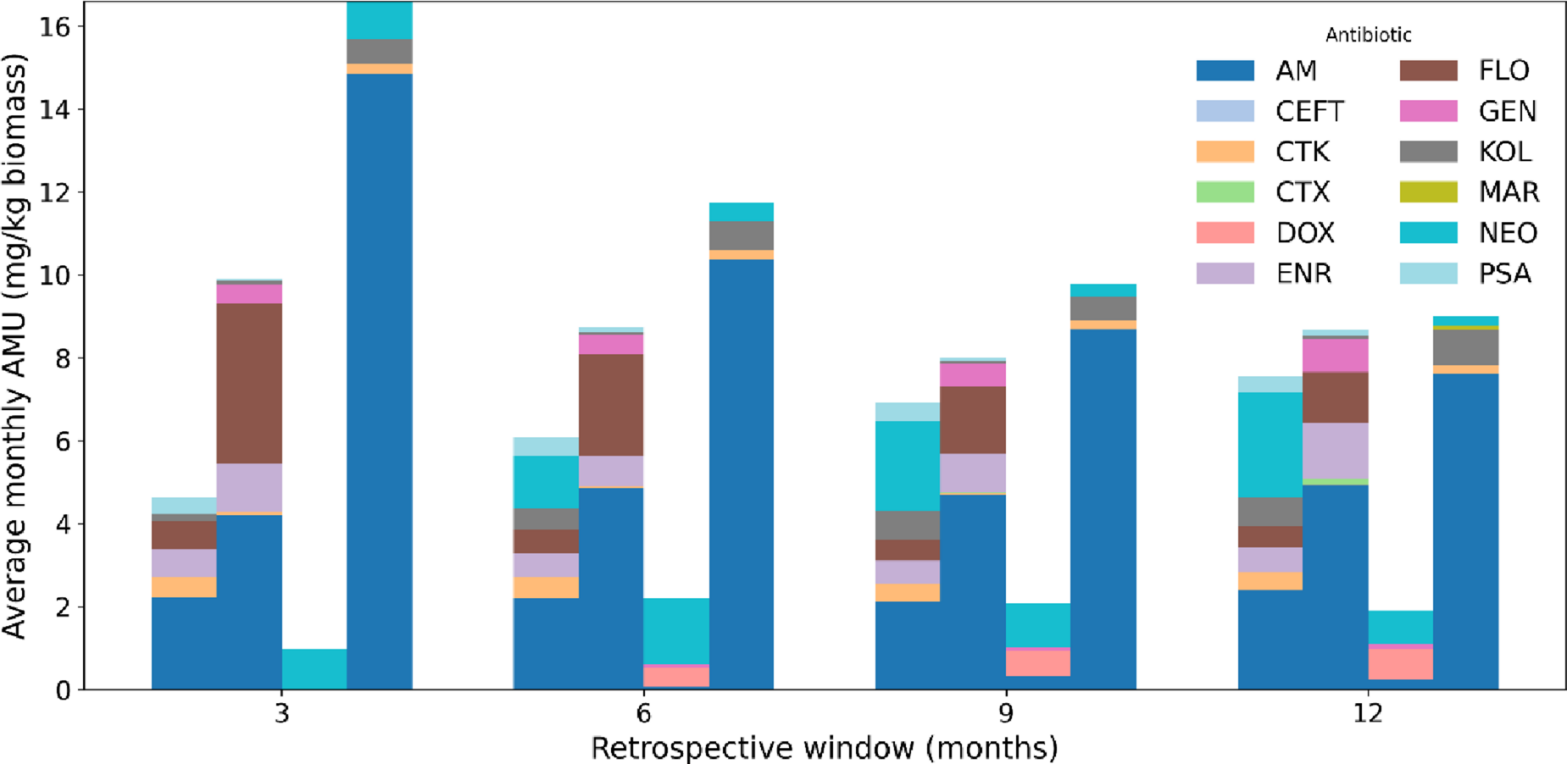
Average monthly antimicrobial use summarized over 3, 6, 9, and 12-month retrospective windows in four farms (1–4 representing the four farms; stacked by antimicrobials). Antimicrobials tested AM (Amoxicillin), CTX (Oxytetracycline), CTK (Chlortetracycline), CEFT (Ceftiofur), DOX (Doxycycline), ENR (Enrofloxacin), FLO Florfenicol, GEN (Gentamicin), KOL (Colistin), MAR (Marbofloxacin), NEO (Neomycin), and PSA (Sulfamethoxazole–Trimethoprim).

AMU varied greatly among the four farms and the observed patterns depended on the length of the retrospective window (Fig. 1). Across all windows, Farm 4 showed the highest (11.77 ± 2.64) average monthly use, Farm 2 was intermediate (8.82 ± 0.60), Farm 1 was lower (6.30 ± 0.98), and Farm 3 consistently had the smallest (1.78 ± 0.42) totals. These between-farm differences remained visible over analyzed 3, 6, 9, or 12 months, retrospectively. For simplicity, Q1, Q2, Q3, and Q4 will be used to represent the 3-, 6, 9-, and 12-month retrospective windows, respectively, and will be referred to throughout the text for clarity.

Time window length revealed different patterns by farm. For Farm 4, average monthly use declined as the window increased, consistent with higher recent use that appears less pronounced when longer time frames are considered. In contrast, Farm 2 was relatively stable as well as Farm 3 in Q2-Q4 and Farm 1 showed slight increase overtime with longer windows (Q3, Q4), indicating comparatively lower use in Q1, Q2 than Q3, Q4. The most dominant antimicrobial (AB) was AM in every farm and time window, except for Farm 3, forming the bulk of each stacked bar. FLO contributed substantially, especially in the shorter windows (Q1, Q2) of Farms 2 and 3, while NEO and PSA were steady but smaller components. CEFT, CTK, CTX, KOL, MAR, DOX, ENR and GEN were present at low levels and appeared episodic (Fig. 1).

### AMR values on each farm

**Fig. 2:**
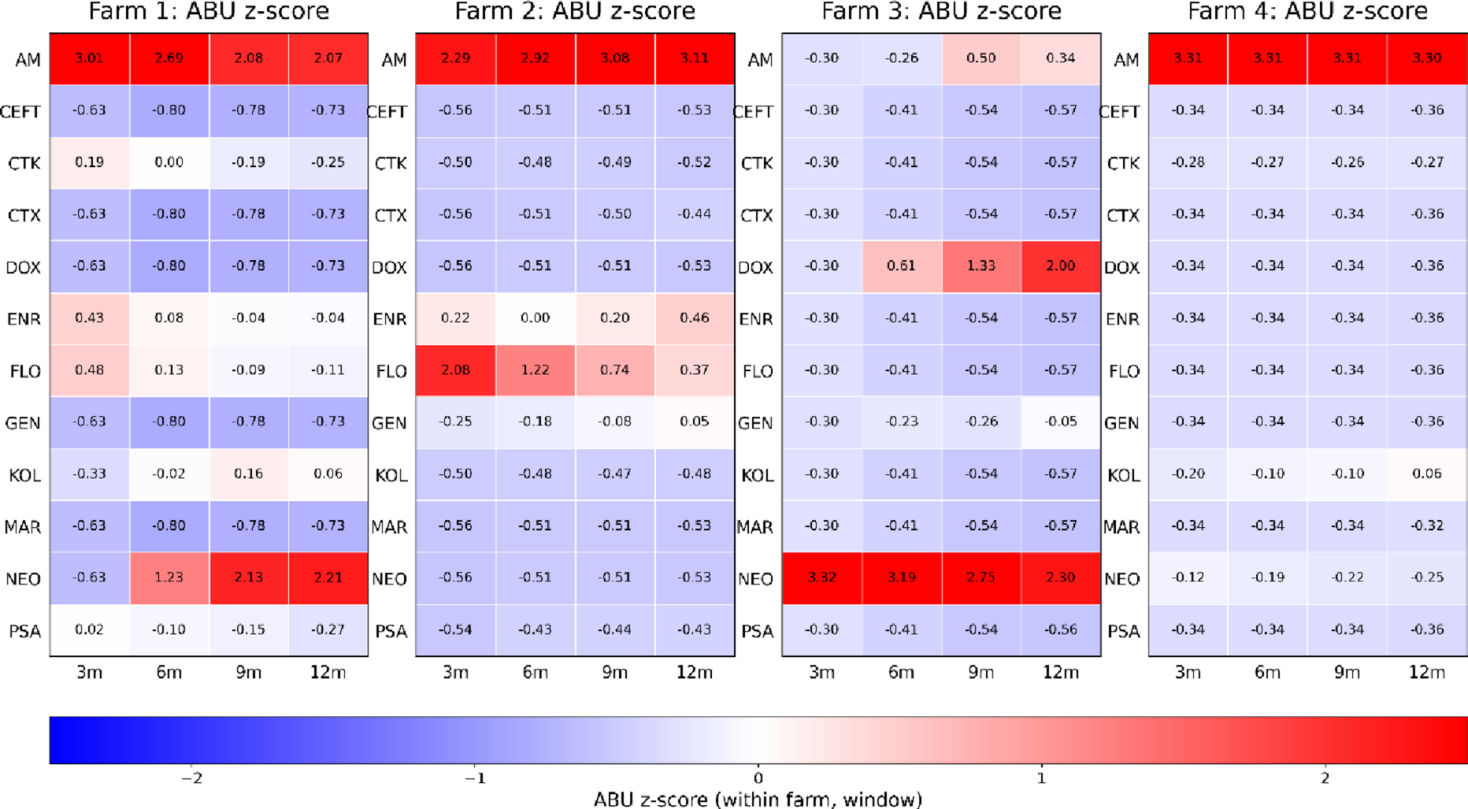
Heatmaps of antimicrobial-specific usage z-scores by farm across 3, 6, 9, and 12-month retrospective windows. Positive values (red) indicate disproportionately higher antimicrobial use than the baseline of farm in that window. Negative values (blue) indicate under-representation. Antimicrobial tested AM (Amoxicillin), CTX (Oxytetracycline), CTK (Chlortetracycline), CEFT (Ceftiofur), DOX (Doxycycline), ENR (Enrofloxacin), FLO Florfenicol, GEN (Gentamicin), KOL (Colistin), MAR (Marbofloxacin), NEO (Neomycin), and PSA (Sulfamethoxazole–Trimethoprim).

Normalized AMU within each farm highlighted over- or under-use in each retrospective window (Fig. 2). Across several farms, AM is consistently over-used relative to the farm’s baseline, with strong positive z-scores in Farms 1, 2, and 4 throughout all windows.

As mentioned briefly Farm 1 was mostly AM-dominant but, unlike Farms 2 and 4, Farm 1 and 3 exhibited a growing contribution of NEO as the window expanded, from mildly positive at Q1 to strongly positive by Q4, suggesting NEO use was more prominent in Q4 than in Q1. ENR and FLO were only modestly positive and most other ABs remain negative in all time windows. Farm 2 is likewise AM-dominant, although showed a secondary signal of FLO use that was highest in Q1-Q2 and attenuated as the window lengthened, consistent with more recent FLO use.

Farm 3 deviated from the other farms. In contrast to other farms, AM in Farm 3 remained close to z = 0, and most remaining ABs consistently showed negative values. However, NEO was a clear outlier with very large positive z-scores in every window, while DOX showed constant increase with longer windows, indicating comparatively greater use in Q3-Q4.

Farm 4 showed the most extreme pattern. AM remained highly positive (z = + 3.31) while nearly all other values were negative across Q1-Q4, indicating a near single-drug profile (Fig. 2).

**Fig. 3:**
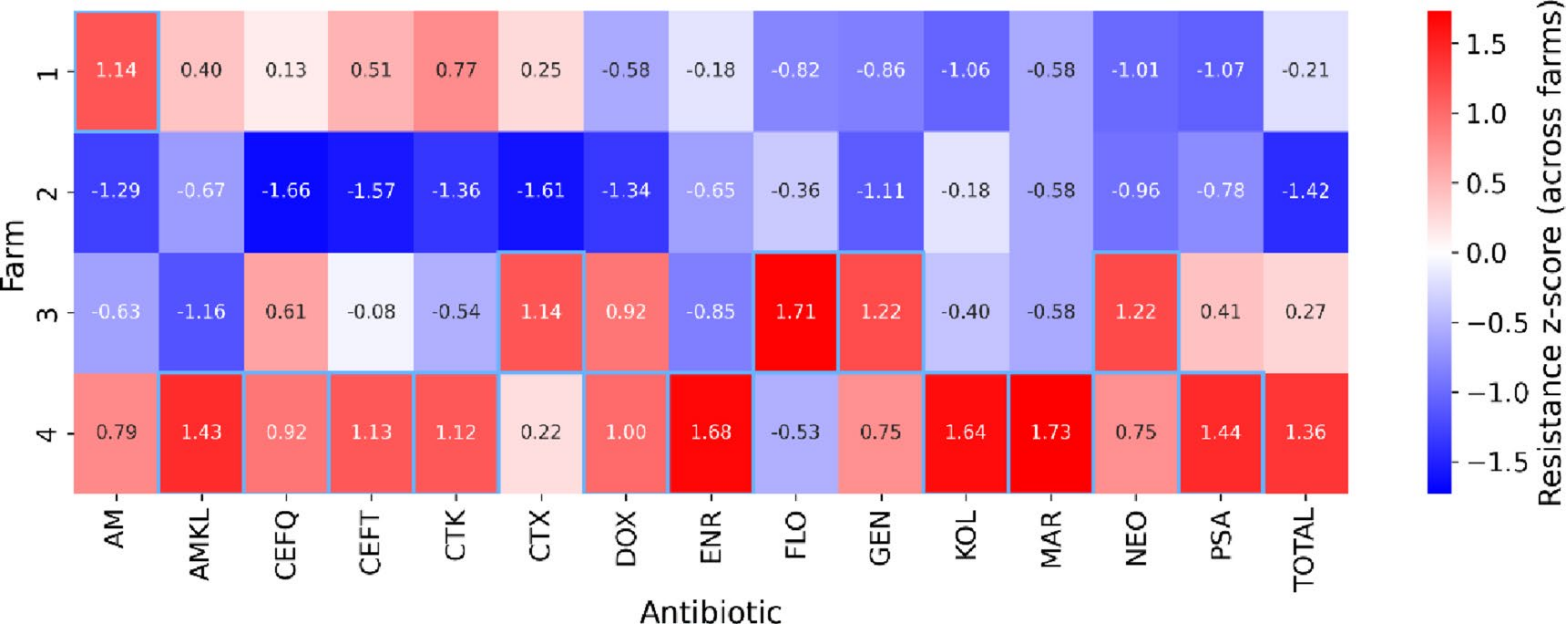
Heatmap of epidemiological AMR z-scores by farm and antimicrobial, including an overall total as a farm-level aggregate. Positive values (red) indicate above-average resistance; negative values (blue) indicate below-average resistance. Antimicrobials tested AM (Amoxicillin) AMKL (Amoxicillin Clavulanic-acid), CTX (Oxytetracycline), CTK (Chlortetracycline), CEFT (Ceftiofur), CEFQ (Cefquinome), DOX (Doxycycline), ENR (Enrofloxacin), FLO Florfenicol, GEN (Gentamicin), KOL (Colistin), MAR (Marbofloxacin), NEO (Neomycin), and PSA (Sulfamethoxazole–Trimethoprim).

Standardized AMR scores varied greatly among farms and across antimicrobials. Total AMR values showed a clear gradient. Farm 4 was highest (z = + 1.36), Farm 3 was slightly above average (z = + 0.27), Farm 1 was slightly below average (z = − 0.21), and Farm 2 was the lowest (z = − 1.42) (Fig. 3).

Farm 1 exhibited a mixed patterns. Higher values were found to AM (z = + 1.14), CTK (z = + 0.77), and CEFT (z = + 0.51), but strong negative ones for FLO (z = − 0.82), GEN (z = − 0.86), NEO (z = − 1.01) and KOL (z = − 1.06), yielding a slightly negative total of − 0.21 (Fig. 3).

Farm 2 showed uniformly lower-than-average values with the strongest negatives at CEFQ (z = − 1.66), CTX (z = − 1.61), CEFT (z = − 1.57), CTK (z = − 1.36), DOX (z = − 1.34), and AM (z = − 1.29), and with a total value of z = − 1.42 (Fig. 3).

Farm 3 was heterogeneous with some higher values for FLO (z = + 1.71), GEN (z = + 1.22), NEO (z = + 1.22), CTX (+ 1.14), DOX (+ 0.92), and CEFQ (z = + 0.61). There were contrasted by negatives for AM (z = − 0.63), ENR (z = − 0.85) and AMKL (z = − 1.16) with a total of near the overall value of z = + 0.21 (Fig. 3).

Farm 4 showed a broadly elevated AMR profile across most antimicrobials (TOTAL z = + 1.36), with the largest positives for MAR (z = + 1.73), ENR (z = + 1.68), KOL (z = + 1.64), PSA (z = + 1.44), AMKL (z = + 1.43), and CEFT (z = + 1.13), CTK (z = + 1.12), DOX (z = + 1.00), CEFQ (z = + 0.92), AM (z = + 0.79), finally GEN and NEO (both + 0.75) among others. Displaying negative values only for FLO (−0.53) (Fig. 3).

**Fig. 4:**
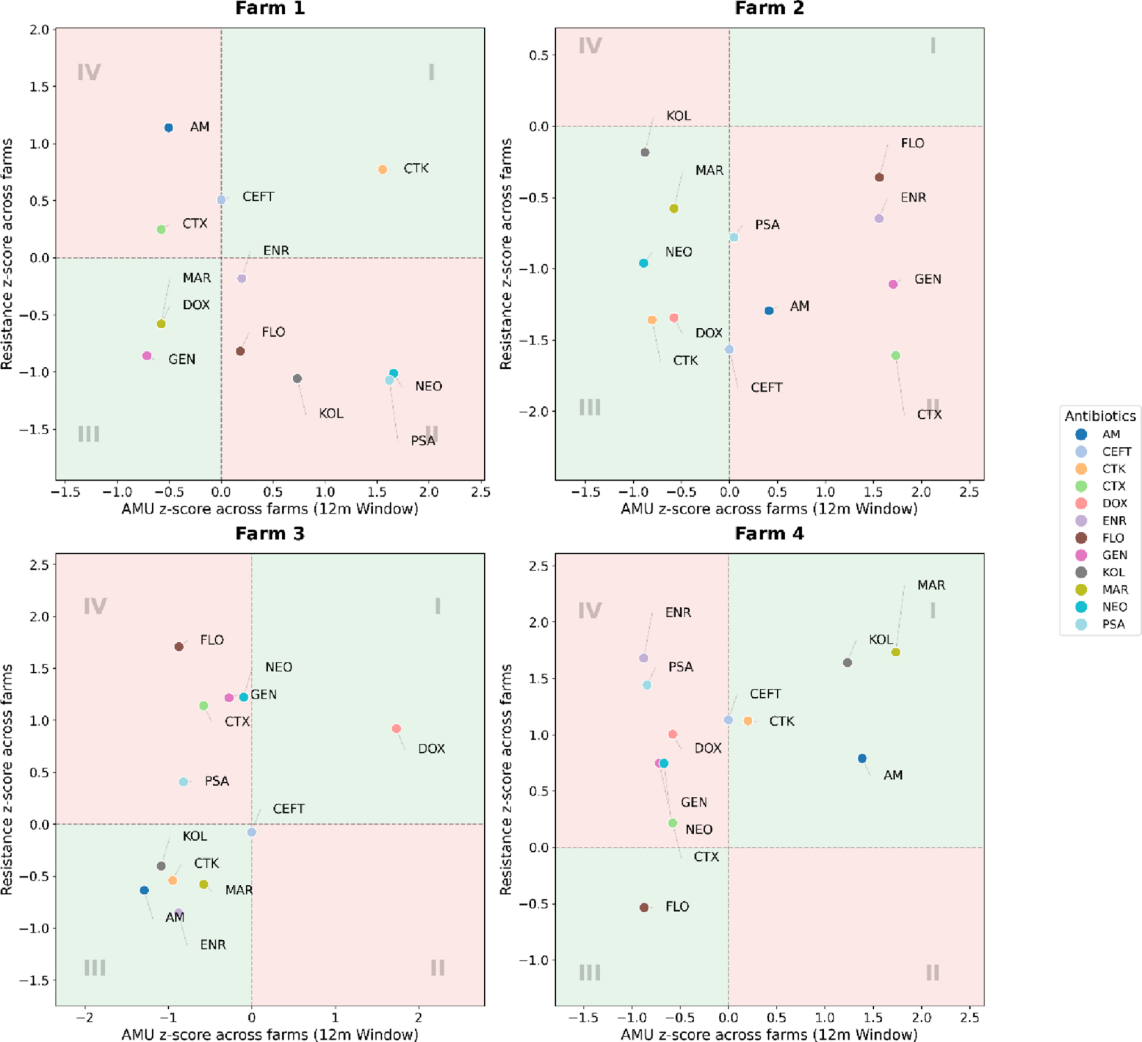
Scatterplots of within-farm z-scores comparing antimicrobial use and AMR for the 12-month window for Farm 1–4. Dashed lines mark the within-farm means (z = 0) for use and AMR. Antimicrobials tested AM (Amoxicillin), CTX (Oxytetracycline), CTK (Chlortetracycline), CEFT (Ceftiofur), DOX (Doxycycline), ENR (Enrofloxacin), FLO Florfenicol, GEN (Gentamicin), KOL (Colistin), MAR (Marbofloxacin), NEO (Neomycin), and PSA (Sulfamethoxazole–Trimethoprim).

Figure 4 displays, for each farm, the z-score of ABU against the z-score of phenotypic resistance within each farm for the 12-month time window, categorized into Agreeing (Quadrants I and III) and Contradicting (Quadrants II and IV) relationships.

Farm 1 showed mixed results. CTK occupied Quadrant I (Agreeing), indicating high use and high resistance values, whereas NEO and PSA fell in Quadrant II (Contradicting) with high use coupled with low resistance. Some other ABs clustered in Quadrant IV (Contradicting), indicating high resistance despite comparatively low recent use, while other ABs fell in Quadrant III (Agreeing) with low use and low resistance.

Farm 2 resistance z-scores showed mostly negative or zero values across ABs. Notably, CTX, GEN, ENR, and FLO showed the highest positive values regarding ABU but remained clearly below zero for resistance, placing them in Quadrant II (Contradicting). No AB appeared in Quadrant I (Agreeing).

Farm 3 showed a clear positive relation for DOX, which was positioned in Quadrant I (Agreeing) with strongly positive use and resistance. In contrast, FLO, GEN, NEO, and CTX showed the opposite pattern in Quadrant IV (Contradicting) with lower use but still higher resistance, while many other AB remained in Quadrant III (Agreeing).

Farm 4 exhibited a pattern where most ABs had high resistance values with near- or below-average use, falling into Quadrant IV (Contradicting). Exceptions included AM, KOL, and MAR with high use and positive resistance values in Quadrant I (Agreeing), whereas only FLO fell into Quadrant II (Contradicting) with resistance below zero.

### Correlation of mean MIC values across farms

**Fig. 5:**
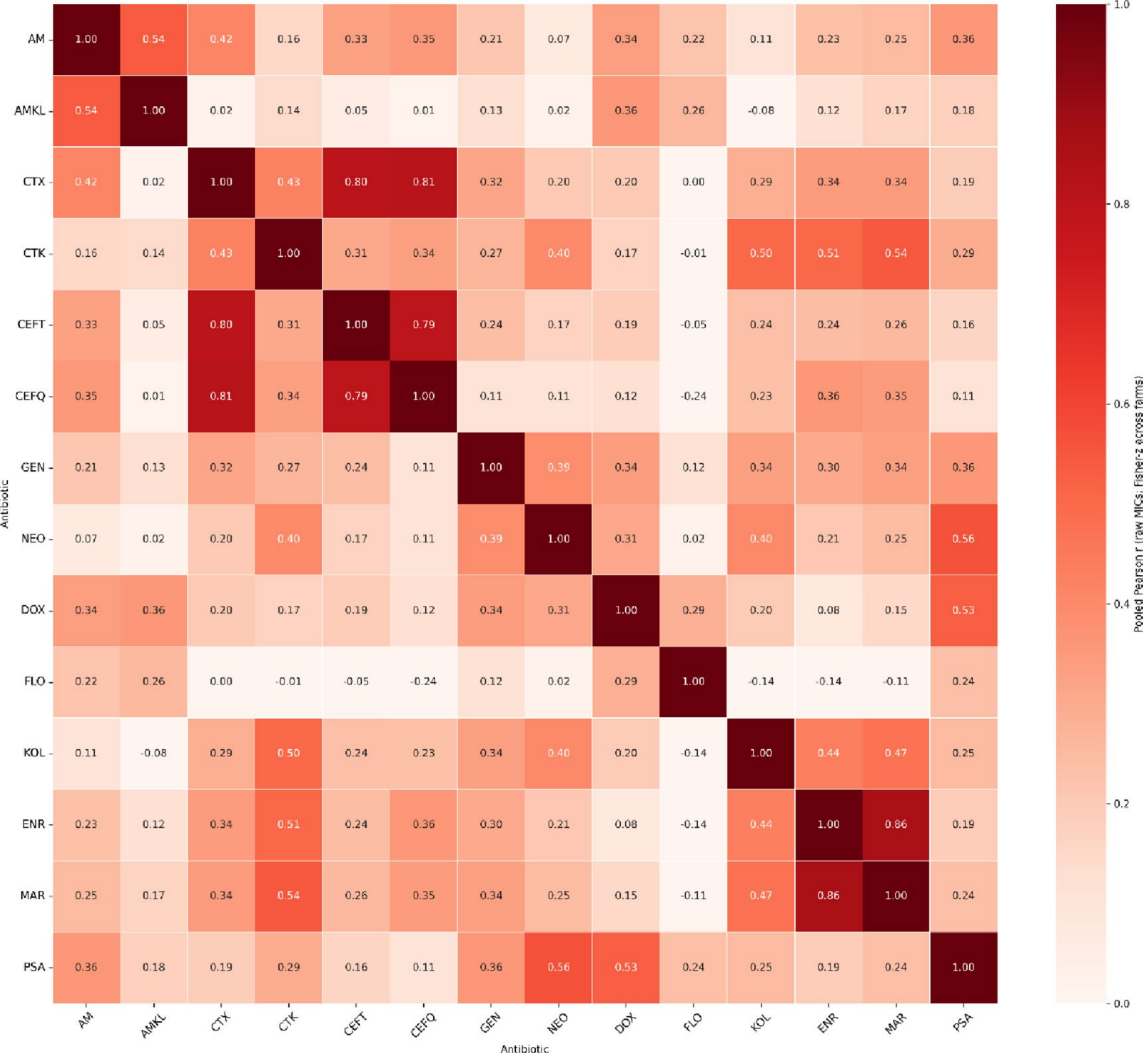
Correlation matrix of minimum inhibitory concentration (MIC) values across antimicrobials and farms (raw isolate MICs, pooled across farms). Each cell shows the pooled Pearson correlation coefficient (r) between two antimicrobials, computed from log2-transformed isolate-level MIC values within each farm (minimum 6 paired isolate measurements per farm) and then combined across the four farms using Fisher’s z fixed-effect meta-analysis. Red shades indicate positive correlations (co-variation of MICs across isolates), while cooler shades indicate weaker or negative correlations. The diagonal (*r* = 1) represents perfect self-correlation. Antimicrobials tested: AM (Amoxicillin), AMKL (Amoxicillin–clavulanic acid), CTX (Cefotaxime), CTK (Chlortetracycline), CEFT (Ceftiofur), CEFQ (Cefquinome), DOX (Doxycycline), ENR (Enrofloxacin), FLO (Florfenicol), GEN (Gentamicin), KOL (Colistin), MAR (Marbofloxacin), NEO (Neomycin), and PSA (Sulfamethoxazole–trimethoprim).

To explore whether susceptibilities to different antimicrobials tend to covary across production units, we quantified pairwise associations between MICs using raw isolate-level data within each farm, then combined evidence across farms using Fisher’s z meta-analysis (Fig. 5). Specifically, for each antimicrobial pair we computed a farm-specific Pearson correlation between log2 (MIC) values across isolates (requiring ≥ 6 paired observations per farm), and then pooled correlations across farms using a fixed-effect Fisher-z model (weights ∝ *n* − 3). Given the small number of farms, we treat these results as hypothesis-generating rather than definitive evidence of co-selection or shared mechanisms, and we computed the between-farm spread of correlations (min–max and SD) alongside heterogeneity diagnostics (e.g., I² and *p* for heterogeneity). The pooled correlation structure highlighted several biologically plausible clusters. Strong positive pooled correlations were observed among fluoroquinolones (ENR–MAR; *r* = 0.857), and among β-lactams (e.g., CTX–CEFQ; *r* = 0.812; CTX–CEFT; *r* = 0.803; CEFT–CEFQ; *r* = 0.794). A moderate positive association was also present between AM and AMKL (*r* = 0.542). We additionally observed positive correlations spanning classes (e.g., DOX–PSA; *r* = 0.527; NEO–PSA; *r* = 0.564), while some pairs were weakly related or near zero (e.g., CTX–FLO; *r* ≈ 0.004). Importantly, several of the strongest pooled correlations also exhibited substantial between-farm heterogeneity (high I² in multiple pairs), indicating that the magnitude, and in some cases even the direction, of associations can vary by farm. Therefore, we interpret the matrix as describing patterns of co-variation in phenotypes across farms, not as proof of shared genetic determinants, and we explicitly refrain from mechanistic claims without supporting genomic or larger-scale validation.

## Discussion

This study revealed pronounced heterogeneity in AMU and AMR patterns across four pig farms, with this variability persisting across multiple retrospective windows.

Only Farm 4 exhibited declining average monthly use as the retrospective window lengthened. In contrast, Farm 2 remained relatively stable, while Farms 1 and 3 showed a slight upward AMU trend over time. Across all farms and timeframes, AM was consistently the dominant antimicrobial, followed by FLO, NEO, PSA, whereas other compounds appeared only sporadically (Fig. 1.). These results are aligned with findings of others in pig production from different countries^29,30^. The epidemiological z-score heatmaps and AMR data reveal coherent yet nuanced patterns between AMU and phenotypic resistance. The highest-use farm (Farm 4) also exhibited the highest aggregate AMR, while the lowest-use farm (Farm 2) consistently showed the lowest AMR (Fig. 3). Drug-specific consistencies further support a usage–resistance linkage. These farm-specific trajectories, together with the time-windowed usage patterns, reveal substantial heterogeneity in both the composition and temporal evolution of drug use, identifying compounds that drive AMR risks within each system. Nevertheless, our results also indicate that AMU and AMR are not always directly aligned. For example, Farm 2 displayed relatively high AM usage but negative AMR z-scores across all antimicrobials, suggesting that high recent use does not necessarily produce immediate increase in AMR. For instance, Pollock et al.^31^ found no measurable increase in AMR gene abundance during a six-month production cycle despite high antimicrobial usage on the farm. Similarly, Smith et al.^32^ and Birkegård et al.^33^ observed that changes in AMU did not necessarily correspond with changes in resistance gene prevalence, indicating a more complex, delayed or even latent relationship between use and AMR. The within-farm scatterplots further highlight these inconsistencies (Fig. 4.). Farm 2 CTX, GEN, ENR, FLO and AM all appeared in the lower-right quadrant (high use but low resistance) contradicting the expected positive correlation. Similar patterns were observed in other farms, where antimicrobials with low current usage still exhibited high AMR values, suggesting the persistence of resistant strains or historical legacy effects. Such findings are again consistent with the concept that the relationship between AMU and AMR is potentially non-linear, time-lagged, and context-dependent^32,34^.

Furthermore, in this study certain antimicrobials showed some associations with resistance outcomes. Notably, high DOX usage on Farm 3 was accompanied by increased resistance, that may indicate a direct selection effect. Similarly, NEO use displayed parallel increases in resistance, and correlation patterns suggest the possibility of cross-resistance within its class. In contrast, AM presented no uniform trend despite being the most frequently used compound across farms. For example, Farm 1 exhibited high AM use and high resistance, whereas Farm 2 showed similarly high use but low resistance levels. This highlights that the role of specific antimicrobials, for example AM, in shaping AMR is likely influenced by farm-specific conditions such as management history, bacterial community structure, disease pressure, and biosecurity, rather than following a simple linear usage–response relationship^35,36^.

Interestingly, several studies have reported positive associations between antimicrobial input at farm or national levels and AMR in commensal E. coli and other bacteria^6,37^. However, these associations are often heterogeneous across antimicrobial classes, bacterial species, and study designs, as also observed here as marked by others^16^. Such variability likely reflects complex selective dynamics influenced by fitness costs, horizontal gene transfer, and environmental reservoirs^38^.

Correlation analyses provided further insight into co-resistance architecture within and across antimicrobial classes. Moderate to strong positive correlations observed among β-lactam antimicrobials (CTX, CEFT, CTK, CEFQ) suggested that resistance to one β-lactam compound may coincides with resistance to others, consistent with their shared mode of action and cross-resistance within this group. Similar intra-class linkages appeared among aminoglycosides (GEN–NEO) and fluoroquinolones (ENR–MAR). Notably, CEFT showed moderate cross-class correlations with ENR and MAR, suggesting that co-selection pressures or mobile genetic elements could link β-lactam and fluoroquinolone resistance genes. This pattern mirrors resistome-level findings reported by Li et al.^39^ who demonstrated co-localization of resistance determinants for multiple antimicrobial classes in swine manure and soil microbiomes. The moderate correlation between AMKL and AM further aligns with their structural similarity and overlapping clinical use^40^.

Conversely, certain antimicrobials such as FLO and KOL displayed weak positive or negative correlations with other classes, that might indicate largely independent AMR mechanisms. This aligns with reports that phenicol resistance often arises through distinct mechanisms, rather than co-location with β-lactams^41,42^. Likewise, colistin resistance is typically mediated by mobilized colistin resistance (MCR-1) gene, which is a plasmid-borne determinant enabling transferable resistance^43^, or chromosomal mutations that evolve separately from other resistance determinants. Although, our correlation results reflect similarities in farm-level MIC patterns but do not, by themselves, demonstrate shared genetic resistance mechanisms or true co-selection, which would require confirmation by genomic analyses and larger-scale longitudinal datasets.

These findings suggests that AMU and AMR are linked, yet their relationship might be modulated by temporal dynamics and farm-specific variables (e.g. management, biosecurity, and treatment practices). Similar descriptive associations between AMU and AMR have been reported previously at herd, regional, and national scales, often using standardized indicators such as mg/PCU or DDDvet and focusing on linear or rank-based correlations^13–15^. The present work demonstrates the feasibility of integrating routinely collected farm treatment records with standardized MIC data into a harmonized, analytical framework. This practical data-engineering step is essential for any future implementation of real-time farm-level surveillance systems. Second, by applying multi-window analyses and correlation structures across antimicrobial classes, the study provides a proof-of-concept that such datasets contain sufficient signal and stability to support more advanced modelling approaches, including time-lagged, causal, and machine-learning-based prediction in subsequent work. Importantly, this study represents one of the first systematic farm-level AMU–AMR integration of this type from Hungary and the wider Central–Eastern European region. Additionally, the value of the present work is not limited to its descriptive results, but lies in establishing a reproducible methodological baseline for future targeted studies, intervention trials, and predictive modelling efforts. The current analysis should be regarded as a necessary foundational step that transforms theoretical concepts of data-driven AMR forecasting into a practically implementable research and surveillance framework.

A key strength of this study lies in the integration of routinely collected farm-level AMU records with standardized MIC data across multiple retrospective windows, providing a reproducible foundation for exploratory AMU–AMR analyses.

Some limitations of this study should be acknowledged. First, antimicrobial usage data were aggregated at the farm level, which may obscure within-farm heterogeneity in treatment practices, disease pressure, and animal group composition. Individual- or pen-level data would enable more precise attribution of antimicrobial exposure to resistance outcomes^44^. Second, antimicrobial resistance was assessed exclusively using phenotypic MIC measurements. While MICs provide valuable clinical and epidemiological information, they do not capture the underlying genomic mechanisms of resistance or the presence of mobile genetic elements, limiting mechanistic interpretation of co-variation patterns^45–50^. Third, due to the observational and cross-sectional design, causality between antimicrobial use and resistance cannot be inferred. Reverse causation is possible, and unmeasured confounders, such as farm management practices, biosecurity measures, vaccination protocols, or environmental reservoirs, may have influenced the observed associations^51–55^. Finally, the small number of farms included in this study (*n* = 4) limits generalizability. The results should therefore be regarded as exploratory and hypothesis-generating rather than representative of Hungarian or Central–Eastern European pig production as a whole^44^.

### Future work

Future research can be structured into feasible short-term extensions and longer-term research goals. In the short term, longitudinal animal-level sampling across production cycles would enable tracking of temporal changes in AMR within herds and thus provide stronger evidence on persistence and turnover of resistance phenotypes. In parallel, expanded environmental sampling of soil, water, and manure could be implemented to better characterize potential external reservoirs of resistance and plausible transmission pathways at farm level.

As a longer-term objective, the integration of whole-genome sequencing and plasmid profiling would allow detailed investigation of the genetic basis of co-resistance and horizontal gene transfer, including the role of mobile genetic elements. Controlled intervention studies, such as reduced antimicrobial use, rotation or cycling schemes, and optimized treatment protocols, could subsequently be designed to directly evaluate how management strategies influence AMR dynamics under field conditions at farm-level. Finally, with sufficiently large, longitudinal and multi-source datasets, more sophisticated and advanced modelling frameworks could be developed to predict how changes in AMU may affect AMR trajectories under different ecological and management scenarios.

## Conclusion

This study demonstrated that farm-level AMU and AMR are meaningfully associated, with these relationships influenced by temporal dynamics and co-resistance. The dominance of AM in both usage and AMR underscores the need to prioritize stewardship strategies that reduce reliance on high-frequency antimicrobials while addressing the cascading effects of cross-resistance across classes. The observed cross-antimicrobial correlations highlight the complexity of AMR ecology and reinforce the importance of integrative, multi-dimensional surveillance systems. Beyond the findings, the current study provides a comprehensive descriptive foundation for future analytical advancements. Upcoming research will focus on applying advanced methodologies, including machine learning to capture complex, non-linear associations and identify key predictive factors, as well as Bayesian modelling to quantify uncertainty and forecast AMR emergence. Together, these approaches may facilitate more precise, data-driven, and evidence-based antimicrobial stewardship strategies in livestock production systems.

## Methods

### Study design and setting

The data that fed this work was achieved from a two-part data collection program that was analyzed as a retrospective, farm-level observational study. The first part was a national digitalization initiative led by Agrofeed Ltd., which has collected routine pig-farm data from multiple enterprises across the country over several years, including AMU from monthly pharmacy records and epidemiological/herd metrics. Product names were standardized and mapped to antimicrobial codes to enable cross-farm, cross-year comparisons. The second part was a targeted sampling for AMR analyses conducted in December 2023 on four commercial pig farms. Microbiological sampling was performed on all four farms, yielding 203 *E. coli* isolates from rectal swabs across age groups. AMU records were available for the four farms covering 2022 February to 2025 January time period. Some information about the farms included in this study is presented in Tabel 1. After receiving harmonizing, and evaluating both databases (Agrofeed Ltd. digitalization dataset and the AMR analyses), AMU-AMR relationship analysis was found to be feasible.

### Ethics and animal welfare

The animals included in this study originated from commercial pig farms operating in Hungary and were privately owned by the respective farm companies. All sampling was conducted on-site as part of routine veterinary diagnostic activities. Written informed consent for sample collection and the use of anonymized farm-level data was obtained from the farm owners or authorized farm managers prior to inclusion in the study. Rectal swab samples were collected from clinically healthy pigs. No animals were subjected to experimental manipulation, additional handling beyond routine veterinary practice, or treatment specifically for the purposes of this research. The study did not involve any invasive procedures other than routine rectal swab sampling, and no animals were subjected to anesthesia, analgesia, sedation, or euthanasia at any stage of the study. All procedures were carried out in accordance with applicable national legislation and institutional animal welfare standards.

The study protocol was reviewed by the Animal Welfare Committee of the University of Veterinary Medicine Budapest, which issued a Certificate of Exemption confirming that the project does not constitute an animal experiment under Act XXVIII of 1998 on Animal Protection and Government Decree No. 40/2013 on animal experiments Table 1.

**Table 1:** Summary of the four swine farm characteristics.

Farms^1^	Farm 1	Farm 2	Farm 3	Farm 4
Production type	Farrow-to-finish	Farrow-to-finish	Farrow-to-finish	Farrow-to-finish
Farm building age	Old	New	Old	New
Health status	Several health problems (e.g. respiratory symptoms, *Actinobacillus pleuropneumoniae*)	Fewer health problems (mainly gastrointestinal related health issues after weaning)	Fewer health problems (mainly gastrointestinal related health issues)	Several health problems (e.g. *Streptococcus suis*)
Average herd size	690	517	478	820
Average no. of suckling piglet	1803	743	296	1194
Average no. of lactating sow	48	17	9	106
Average no. of pregnant sow	11	8	23	80
Average no. of pregnant breeding gilt	–	–	10	157
Average no. of breeding gilt prior to insemination	166	–	39	168
Average no. of weaned pig	907	440	723	1206
Average no. of finishing pig	142	1039	–	–

^1^ All farms had PRRS free status

### Data cleaning and preprocessing

#### Antimicrobial usage (AMU) data

AMU data from the database of Agrofeed Ltd. contained monthly farm pharmacy records for each farm, calendar year and month, target age group/herd unit, product/substance name, and quantity. Product names were normalized (lower-cased, Unicode dash harmonization) and mapped to antimicrobial codes used in the MIC panel via a predefined dictionary (e.g., *amoxicillin-trihydrate* → AM; *enrofloxacin* → ENR) (see Sect. 2.2.2).

To align exposure with outcomes, AMU for each farm and antimicrobial was aggregated over retrospective windows of 3, 6, 9, and 12 months immediately preceding the sampling month for that farm. For each window, the quantities were summed and divided by the window length to obtain the average monthly usage.

### MIC analyses data

Rectal swab samples were collected across age groups (newborn, 4-week-old, 6-week-old). *E. coli* was isolated and tested for MICs against 14 agents: AM, AMKL, CTX, CTK, CEFT, CEFQ, GEN, NEO, DOX, FLO, KOL, ENR, MAR, PSA. MICs were recorded as numeric dilution values (mg/L). Binary resistance calls were derived using two breakpoint systems for each antimicrobial: a clinical breakpoint^56^, and, where it was not available, an *epidemiological cutoff (ECOFF)*^57^. A sample was classified as resistant if *MIC ≥ cutoff*. The cutoffs used in the analysis were (ECOFF; clinical):

- AM (Amoxicillin): 8; 32.
- AMKL (Amoxicillin–Clavulanic Acid): 8; 32.
- CTX (Oxytetracycline): 0.25; 0.25.
- CTK (Chlortetracycline): 0.25; 0.25.
- CEFT (Ceftiofur): 1; 8.
- CEFQ (Cefquinome): 0.125; 2.
- GEN (Gentamicin): 2; 8.
- NEO (Neomycin): 8; 8.
- DOX (Doxycycline): 4; 16.
- FLO (Florfenicol): 16; 16.
- KOL (Colistin): 2; 2.
- ENR (Enrofloxacin): 0.125; 2.
- MAR (Marbofloxacin): 4; 4.
- PSA (Sulfamethoxazole–Trimethoprim): 0.5; 4.

### Data processing

AMU was normalized to mg per kg of livestock biomass, computed from each farm’s own inventory rather than ESVAC/EMA national proxies. Concretely, for each farm and calendar month we derived the biomass as the headcount × average weight at treatment summed over age/production classes recorded on site (e.g., suckling piglets, weaners/growers, finishers, sows). Average weights were the farm-level operational weights used in routine records (sector-typical values, e.g., ~ 3.5 kg for suckling piglets, ~ 25 kg for weaners, ~ 65 kg for finishers, ~ 240 kg for sows), and the livestock counts reflected the weekly batching, farrow-to-finish production used by all participating farms. Using these inventory-based denominators yields a modified mg/kg biomass metric that is directly aligned with the farms’ production flows rather than with country-level ESVAC assumptions. Let ${D}_{fa,t}$ denote the mg of active ingredient dispensed for farm $f$, antimicrobial $a$, in month $t$. Let the biomass for farm $f$ in month $t$ be

1 $${B}_{f,t}{\hspace{0.25em}\hspace{0.05em}}={\hspace{0.25em}\hspace{0.05em}}\sum_{c}{N}_{f,c,t}\times{\mathrm{A}\mathrm{W}\mathrm{T}}_{c},$$

where ${N}_{f,c,t}$ is the inventory count in class $c$ and ${\mathrm{A}\mathrm{W}\mathrm{T}}_{c}$ is the class-specific average weight at treatment as Eq. (1). The monthly AMU density is then

2 $${M}_{fa,t}{\hspace{0.25em}\hspace{0.05em}}={\hspace{0.25em}\hspace{0.05em}}\frac{{D}_{fa,t}}{{B}_{f,t}}mg/kg\;biomass$$

was calculated as in Eq. (2). To align exposure with outcomes, for each farm $f$, antimicrobial $a$, and retrospective window $w\in\{\mathrm{3,6},\mathrm{9,12}\}$ months immediately preceding that farm’s December-2023 sampling (exclusive), we computed the average monthly AMU as

3 $$U_{{fwa}} = \frac{1}{w}\sum\limits_{{t\varepsilon W\left( w \right)}} {M_{{fa,t}} } \left( {mg/kg\;biomass\;per\;month} \right),$$

where $\mathcal{W}\left(w\right)$ indexes the months in the $w$-month look-back. Before any normalization, AMU was expanded to the full farm × window × antimicrobial grid and zero-imputed where records were absent (interpreted as no use) as in Eq. (3). This prevents sparsity from inflating standardized values and ensures that true non-use contributes as negative standardized scores rather than appearing as missing. Within each farm and window, we then standardized AMU across antimicrobials to characterize the farm’s internal usage mix. *A* denote the number of antimicrobial observed (after zero-imputation) at farm *f* in window *w*. The within-farm AMU z-score is:

4 $${z}_{fwa}^{ABU}=\frac{{U}_{fwa}-{\mu}_{fw}}{{\sigma}_{fw}},{\mu}_{fw}=\frac{1}{A}\sum_{a}{U}_{fwa},{\sigma}_{fw}=\sqrt{\frac{1}{A}\sum_{a}{({U}_{fwa}-{\mu}_{fw)}}^{2}}$$

If ${\sigma}_{fw}=0$ (i.e., identical values across antimicrobials), we set ${z}_{fwa}^{ABU}=0$ to avoid division by zero as in Eq. (4) These z-scores are used in the AMU heatmaps (by farm and window) and as the x-axis in the 12-month farm-specific scatterplots (Fig. 4). For resistance, we computed, per farm *f* and antimicrobial *a*, the resistance rate ${R}_{fa}$​ as the mean of the resistant indicator (MIC ≥ breakpoint) across all *E. coli* isolates from that farm, under both ECOFF and clinical breakpoints (cutoff values listed above). To permit fair between-farm comparisons for a given antimicrobial, we standardized across farms:

5 $${z}_{fa}^{RES}=\frac{{R}_{fa}-{\mu}_{a}}{{\sigma}_{a}},{\mu}_{a}=\frac{1}{F}\sum_{f}{R}_{fa},{\sigma}_{a}=\sqrt{\frac{1}{F}\sum_{f}(}{{R}_{fa}-{\mu}_{a})}^{2}$$

where F is the number of farms with AMR data for antimicrobial *a*. When ${\sigma}_{a}=0$, we set ${z}_{fa}^{RES}=0$. These across-farm AMR z-scores populate the AMR heatmap and provide the y-axis for the 12-month scatterplots as in Eq. (5).

As a supplementary summary of phenotypic structure, we assessed co-variation among antimicrobial MICs by correlating isolate-level MIC measurements between AB pairs. MICs were first transformed (6) to the doubling-dilution scale,

6 $${x}_{ifa}={\mathrm{log}}_{2}\left(MI{C}_{ifa}\right)$$

For each farm *f* and AB pair *(a*,* b)*, we computed a farm-specific Pearson correlation ${r}_{f}\left(a,b\right)$ across isolates with both MICs available; pairs were retained only if at least ${n}_{min}=6$ isolate pairs were present within that farm. To summarize correlations across farms, we applied Fisher’s *z*-transformation (7):

7 $${z}_{f}={\mathrm{tanh}}^{-1}\left({r}_{f}\right)$$

and pooled farm-specific estimates (8) using inverse-variance weights ${\omega}_{f}={n}_{f}-3$ (fixed-effect meta-analysis):

8 $${z}_{FE}={\sum}_{}^{}{\omega}_{f}{z}_{f}/{\sum}_{}^{}{\omega}_{f},with\;{r}_{FE}=\mathrm{tanh}\left({z}_{FE}\right)$$

Two-sided p-values and 95% confidence intervals were computed on the z-scale and back-transformed to *r*. To reflect the small number of farms and potential between-farm variability, we additionally reported the spread of ${r}_{f}$ values and, where enabled, provided sensitivity analyses using random-effects pooling and a cluster bootstrap by farm (resampling farms with replacement) to obtain robust empirical confidence intervals. The final pooled correlation matrix was visualized as a heatmap (diagonal = 1).

### Evaluation and statistical approach

All analyses were performed in Python 3.10. Data handling used pandas and NumPy, statistical tests used SciPy, and visualizations were produced with Matplotlib and Seaborn, with one interactive figure rendered using Plotly. File I/O and paths used the Python standard library (os, pathlib). Exact package versions are provided in the project environment report. Descriptive statistics were used to summarize AMU and AMR across farms and antimicrobials. Standardized z-scores, computed as (value - mean)/standard deviation, were used as unit-free indicators to evaluate relative deviations from the respective farm- or antimicrobial-specific means. While z-scores facilitate relative comparisons, we acknowledge potential interpretive challenges, such as distortions in ratios or assumptions of unimodal distributions, particularly in small samples^58^. These standardized values were visualized in heatmaps to outline antimicrobial-specific usage and resistance patterns across the four retrospective windows (3, 6, 9, and 12 months). To examine within-farm associations between AMU and AMR, bivariate scatterplots were generated for the 12-month window, plotting AMU z-scores against the corresponding AMR z-scores, with quadrants labelled to highlight patterns (e.g., upper-right: high use–high resistance). Potential co-resistance relationships were explored by calculating pairwise Pearson correlation coefficients between antimicrobials using log2-transformed raw isolate MIC values within each farm, followed by pooling farm-specific correlations via Fisher’s z fixed-effect meta-analysis (weighted by n_f − 3). This provided pooled estimates with 95% confidence intervals and p-values, leveraging the full dataset (*N* = 203 isolates) rather than farm-level means. The resulting correlation matrix was visualized as a diverging color heatmap, with positive values indicating co-variation patterns and negative values indicating independence. Correlation strength was interpreted as weak (|r| < 0.3), moderate (0.3 ≤ |r| ≤ 0.7), or strong (|r| > 0.7), and statistical significance was set at *p* < 0.05; however, given the exploratory nature and small number of farms (*n* = 4), correlations are treated as hypothesis-generating only, without implying robust co-selection or shared mechanisms. Due to the small number of farms (*n* = 4), no inferential modelling or hypothesis testing was applied; the analysis instead focused on standardized descriptive and correlational approaches. The primary outcomes, defined per farm and antimicrobial, included (i) the mean MIC and (ii) the AMR rate under both epidemiological cut-off values (ECOFF) and clinical breakpoint definitions, while secondary outcomes covered pairwise co-variation patterns and within-farm MIC co-variation across antimicrobials. The overarching goal of this preliminary phase was to systematically explore the relationships between antimicrobial usage and AMR patterns, and to assess the feasibility of applying advanced analytical methods, including machine-learning-based predictive frameworks, to these integrated data sources.

## Data Availability

The datasets generated during and analyzed during the current study are not publicly available due to the inclusion of sensitive commercial information from producers but are available from the corresponding author on reasonable request.
